# Extensive identification of serum metabolites related to microbes in different gut locations and evaluating their associations with porcine fatness

**DOI:** 10.1111/1751-7915.14245

**Published:** 2023-03-14

**Authors:** Qin Liu, Maozhang He, Zhijun Zeng, Xiaochang Huang, Shaoming Fang, Yuanzhang Zhao, Shanlin Ke, Jinyuan Wu, Yunyan Zhou, Xinwei Xiong, Zhuojun Li, Hao Fu, Lusheng Huang, Congying Chen

**Affiliations:** ^1^ National Key Laboratory for Swine Genetic Improvement and Production Technology Jiangxi Agricultural University Nanchang China; ^2^ Department of Microbiology, School of Basic Medical Sciences Anhui Medical University Hefei China; ^3^ Research Center for Differention and Development of TCM Basic Theory, Jiangxi Province Key Laboratory of TCM Etiopathogenisis Jiangxi University of Chinese Medicine Nanchang China

## Abstract

Gut microbiota plays important roles in host metabolism. Whether and how much the gut microbiota in different gut locations contributes to the variations of host serum metabolites are largely unknown, because it is difficult to obtain microbial samples from different gut locations on a large population scale. Here, we quantified the gut microbial compositions using 16S rRNA gene sequencing for 1070 samples collected from the ileum, cecum and faeces of 544 F6 pigs from a mosaic pig population. Untargeted metabolome measurements determined serum metabolome profiles. We found 1671, 12,985 and 103,250 significant correlations between circulating serum metabolites and bacterial ASVs in the ileum, cecum, and faeces samples. We detected nine serum metabolites showing significant correlations with gut bacteria in more than one gut location. However, most metabolite‐microbiota pairwise associations were gut location‐specific. Targeted metabolome analysis revealed that CDCA, taurine, L‐leucine and N‐acetyl‐L‐alanine can be used as biomarkers to predict porcine fatness. Enriched taxa in fat pigs, for example *Prevotella* and *Lawsonia intracellularis* were positively associated with L‐leucine, while enriched taxa in lean pigs, such as *Clostridium butyricum*, were negatively associated with L‐leucine and CDCA, but positively associated with taurine and N‐acetyl‐L‐alanine. These results suggested that the contributions of gut microbiota in each gut location to the variations of serum metabolites showed spatial heterogeneity.

## Graphical abstract

In this study, we used a mosaic pig population raised in a uniformed farmhouse as experiment pigs and found significant correlations between circulating serum metabolites and bacterial ASVs in the ileum, cecum, and feces samples. We indicated that most metabolite‐microbiota pairwise associations are gut location‐specific. We further identified four metabolites that can be used for discriminating the pigs with high‐fat deposition. Significant associations between the fatness‐associated bacterial taxa and the fatness‐associated metabolites suggested the possible mechanisms of the gut microbiome affecting host fat deposition.

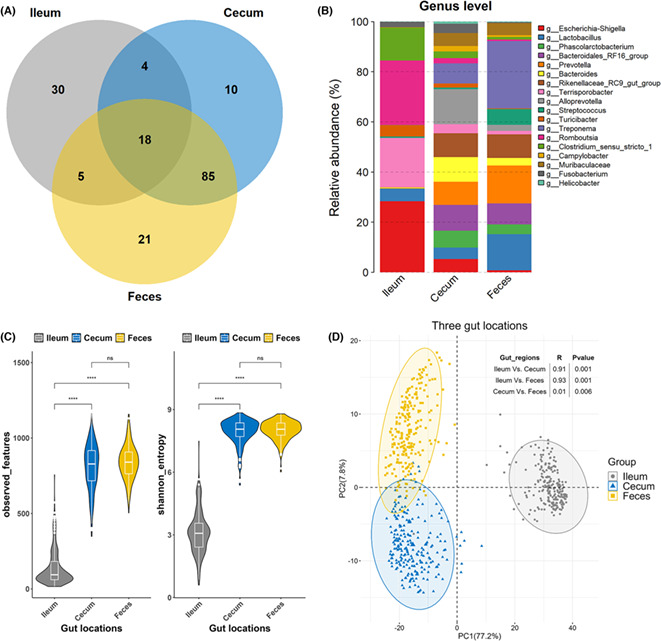

## INTRODUCTION

The gut of pigs harbours a diverse and complex community system mainly formed by a vast amount of gut microbiota. These microbiotas and derived microbial compounds have been proven to play essential roles in host health and diseases. Dysbiosis of the gut microbiota connects with various diseases, such as obesity (Liu et al., 2017; Turnbaugh et al., 2006), insulin resistance (Pedersen et al., 2016) and colorectal cancer (Long et al., 2019). Accumulated evidence in the literature has shown that underlying metabolite features of the fundamental gut microbial taxa determine the production of metabolites that mediate a profound linkage to host‐microbiota communication. The gut microbiome is highly variable and can be shaped by host factors, such as genetics (Yang et al., 2022) and age (Ke et al., 2019), and environmental elements, for example, diet and drugs (Goodrich et al., 2014; Mokkala et al., 2021; Nicolucci et al., 2017). Furthermore, the host immune system also has a great impact on the microbial ecosystem, as well as intestinal metabolic products (Kau et al., 2011).

Metabolites produced by gut bacteria can be partially released into the host's circulation (McFall‐Ngai, 2008; Sharpton et al., 2019). There are up to one‐third of metabolites in the blood that can be derived from gut bacteria (Hood, 2012; Wikoff et al., 2009). Indeed, serum levels of bile acids (BAs), short‐chain fatty acids (SCFAs) (Duscha et al., 2020; Morrison & Preston, 2016), branch chain amino acids (BCAAs) (Newgard et al., 2009; Pedersen et al., 2016), aromatic amino acids (AAAs) and some vitamins (Alexeev et al., 2018) are partly mediated by the metabolisms of host or gut microbiota, or both (Hofmann, 2009). These metabolites have been identified to be related to host metabolic health and/or disorders, such as obesity and insulin sensitivity (Bar et al., 2020; Liu et al., 2017; Pedersen et al., 2016; Turnbaugh et al., 2006). For example, intestinal BAs modified by gut bacteria can exert hormone‐like functions through activating nuclear and membrane‐bound receptors, and thereby modulate host physiological functions, such as glucose, lipid and energy metabolism, growth, intestinal integrity and immunity (Chen et al., 2020; Hofmann, 2009; Wahlstrom et al., 2016). Additionally, phenylacetic acid, a microbially derived byproduct of phenylalanine metabolism, was demonstrated to successfully induce liver steatosis and trigger BCAAs metabolism (Hoyles et al., 2018).

Most of the previous studies designed to assess the contribution of gut microbiota to host metabolites or concerning microbiota‐derived metabolites used faeces as representative and primary samples for the gut microbial composition analyses. Notably, the gut microbial composition and function, metabolomic landscape and host‐microbiota interplay between different anatomical sections of the niche (gut locations) are quite different along with the intestinal compartments, which are caused by peristalsis, pH value, nutrient concentrations, physiological architecture, chemical gradients, oxygen content and host immunity (Hao & Lee, 2004; Kau et al., 2011). Nevertheless, the relationship between serum metabolites reflecting the host physical state and bacterial variations in discrepant gut regions caused by spatial heterogeneity remains unclear.

Of note, the variations in gut microbes induce metabolic shifts that may lead to host physiology alterations. For example, accumulated researches in humans and mice have determined the pivotal role of the gut microbiome in contributing to host obesity (Liu et al., 2017; Mokkala et al., 2021; Nicolucci et al., 2017) by integrated analyses of metagenomic sequencing, single bacterium or prebiotic cocktail colonization of germ‐free mice, and cohousing of mice harbouring different gut microbiota. In pigs, backfat thickness is also a multifactorial and complex porcine fatness trait, and it is influenced by host genetics, nutrition, management and gut microbiota that was documented recently (Yang et al., 2016). The gastrointestinal structure and clinical manifestation of pigs are highly similar to that of humans (Zhang et al., 2013); therefore, pigs are now an optimal model for investigating a variety of human disorders, including obesity and metabolic syndrome. In addition, pigs tend to deposit excess fat and the diets of pigs are readily managed (Zhang et al., 2013): domestic pigs have been regarded as an appropriate animal model for investigating the causal role of gut microbiota in host obesity and fat deposition, and for elucidating the mechanism of gut microbiota in affecting host phenotypes by integrating metabolome and other multi‐omics data. Our previous studies have demonstrated the crucial role of gut microbiota on feed efficiency, fat deposition and blood glucose metabolism in pigs (Huang et al., 2017; Yang et al., 2016, 2017).

Metabolome can be determined by both untargeted and targeted methods. The untargeted metabolome technique measures the metabolites in samples with comprehensive coverage and has contributed to numerous discoveries of microbiota‐related metabolic pathways and metabolites implicated in host health and diseases (Nemet et al., 2020). However, targeted metabolome approaches focus on validating well‐defined groups of metabolites with high specificities, detection sensitivity and quantitative accuracy (Lloyd‐Price et al., 2019; Nicolucci et al., 2017). In many studies, untargeted metabolome analysis was used to identify the relationships between metabolites and microbiota or phenotypes. Then, targeted metabolome analysis was used for validation and quantification analysis.

Here, to explore the relationships between serum metabolome profiles and microbial compositions in different gut locations, we used 544 pigs from an eight‐breed cross heterogeneous population as experimental animals, from which 522 faeces, 300 cecum content, and 248 ileum content samples were taken and their bacterial composition was determined by 16S rRNA gene sequencing. In addition, 544 serum samples from these pigs were used to determine the metabolome profiles. Based on the results of association analyses between microbial taxa and metabolite features, we found a myriad of correlations which included intestinal site‐specific correlations and overlapped correlations across gut locations. Then, we quantified a panel of informative and illuminating metabolites by targeted metabolome analysis. Notably, we presented several instructive metabolite biomarkers that could be used for distinguishing swine with distinct fatness (backfat thickness) at a higher diagnostic rate. This work provided a reference of the relationships between the microbiota from different gut locations and fasting serum metabolites in pigs, and suggested the possibility of monitoring, diagnosing and even treating microbiome‐related fatness or intestinal dysbacteriosis through an easy‐to‐implement, and reliable clinical test with blood samples.

## RESULTS

### The overall landscape of gut microbial compositions of three gut locations and the profiles of serum metabolome in experimental pigs of the F_6_ population

**FIGURE 1:**
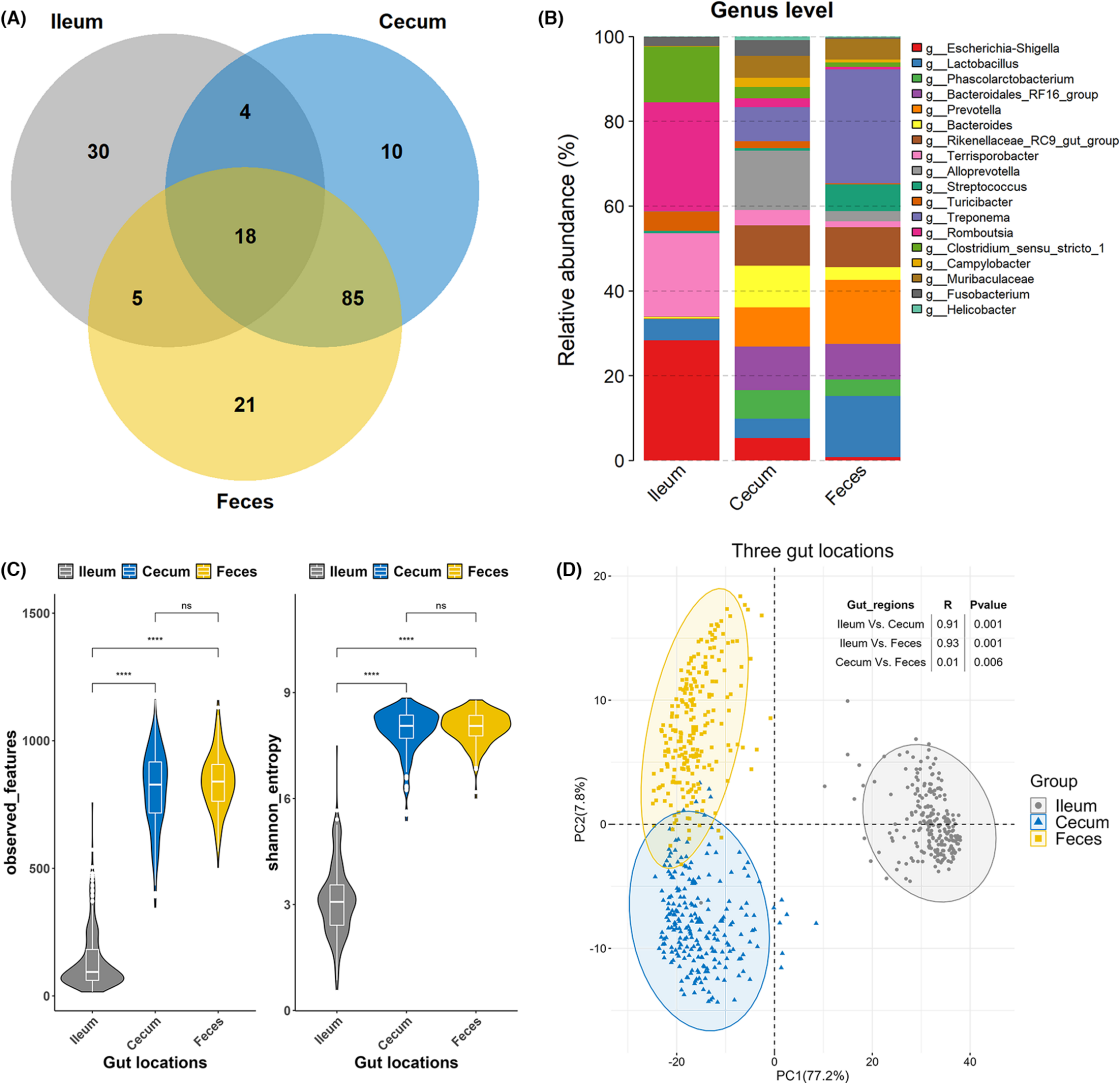
Comparison of microbial compositions across three gut locations. (A) Venn diagram representing the numbers of the shared‐ or gut‐specific microbiota at the genus level. (B) Stacked bar plot showing the relative abundance of 19 core microbes at the genus level. (C) Comparison of alpha‐diversity across different intestinal compartments in 229 pigs with microbial samples from all three gut locations. (D) Principal component analysis based on the Bray–Curtis dissimilarity distance matrix across the ileum, cecum, and faeces in 229 pigs (the inside panel at the top right shows the ANOSIM analysis among different gut locations). The Kruskal‐Wallis test and unpaired Wilcoxon rank‐sum test were performed, and ns means that no significant difference was found between the two groups, ** and *** mean significance levels of *p* < 0.01 and *p* < 0.001.

To decipher the gut microbial composition of experimental pigs, a total of 1070 samples were analysed by 16S rRNA gene sequencing, including 248 ileum content, 300 cecum content and 522 faeces samples. There were 229 pigs from which we collected microbial samples from all three gut locations (Figure S1A). After quality control, a total of 39,253,254 assembled clean tags were obtained and used for further analyses. The number of clean tags per sample ranged from 30,001 to 44,994 with an average number of 36,685. A total of 13,331 amplicon sequence variants (ASVs) were obtained from all tested samples at the 99% sequence identity. Those ASVs with relative abundance >0.05% and identified in more than 5% of individuals were used for subsequent analyses. As a result, 75, 391, and 404 ASVs were retained for ileum, cecum, and faeces samples, respectively (Figure S1B). Taxonomic annotation with the SILVA (v138.1) detected 15 phyla, 131 genera, and 182 species in all tested samples from three gut locations. In more detail, 11 phyla, 13 classes, 34 orders, 41 families, 51 genera and 74 species were identified in 248 ileum samples; 15 phyla, 22 classes, 42 orders, 61 families, 117 genera and 158 species in 300 cecum samples; and 15 phyla, 23 classes, 46 orders, 69 families, 129 genera and 172 species in 522 faeces samples. Moreover, Venn diagram analysis showed 18 core bacterial genera across ileum, cecum and faeces samples (Figure 1A). In addition, *Escherichia‐Shigella*, *Romboutsia* and *Terrisporobacter* were dominant in the ileum content, *Alloprevotella*, *Bacteroidales_RF16_group*, and *Bacteroides* were the three most abundant genera in the cecum content, while *Treponema*, *Prevotella* and *Lactobacillus* were mainly enriched in faeces (Figure 1B). Compared with that in ileum samples, the α‐diversity of the microbial community based on the observed features and Shannon index was significantly increased in cecum and faeces samples in the 229 pigs with microbial samples in all three gut locations (Figure 1C). Divergent compositions of gut microbiota among three gut locations were also observed by PCA analysis in these 229 pigs (Figure 1D).

Untargeted global metabolome profiling was used to quantify the overall composition of serum metabolites. A total of 544 serum samples from this F_6_ pig population were subjected to metabolome measurement. After peak alignment, peak picking and deconvolution, we detected a total of 12,700 and 5423 precursors of m/z values in positive and negative ion modes. We excluded those metabolite features for which the percentage of relative standard deviation (RSD%) was more than 30% in QC samples. The peaks that were presented (non‐zero value) in more than 50% of tested samples were included in further analyses. Ultimately, 3572 and 1794 metabolite features in positive and negative ion modes were reserved, and 128 metabolites were identified based on the annotations with online databases (HMDB and Metlin) and used for subsequent association analysis (Table S1).

### A large quantity of serum metabolites showed significant correlations with the microbiota compositions of ileum, cecum and faeces samples

#### Associations between ileum microbiota composition and serum metabolite features

**FIGURE 2:**
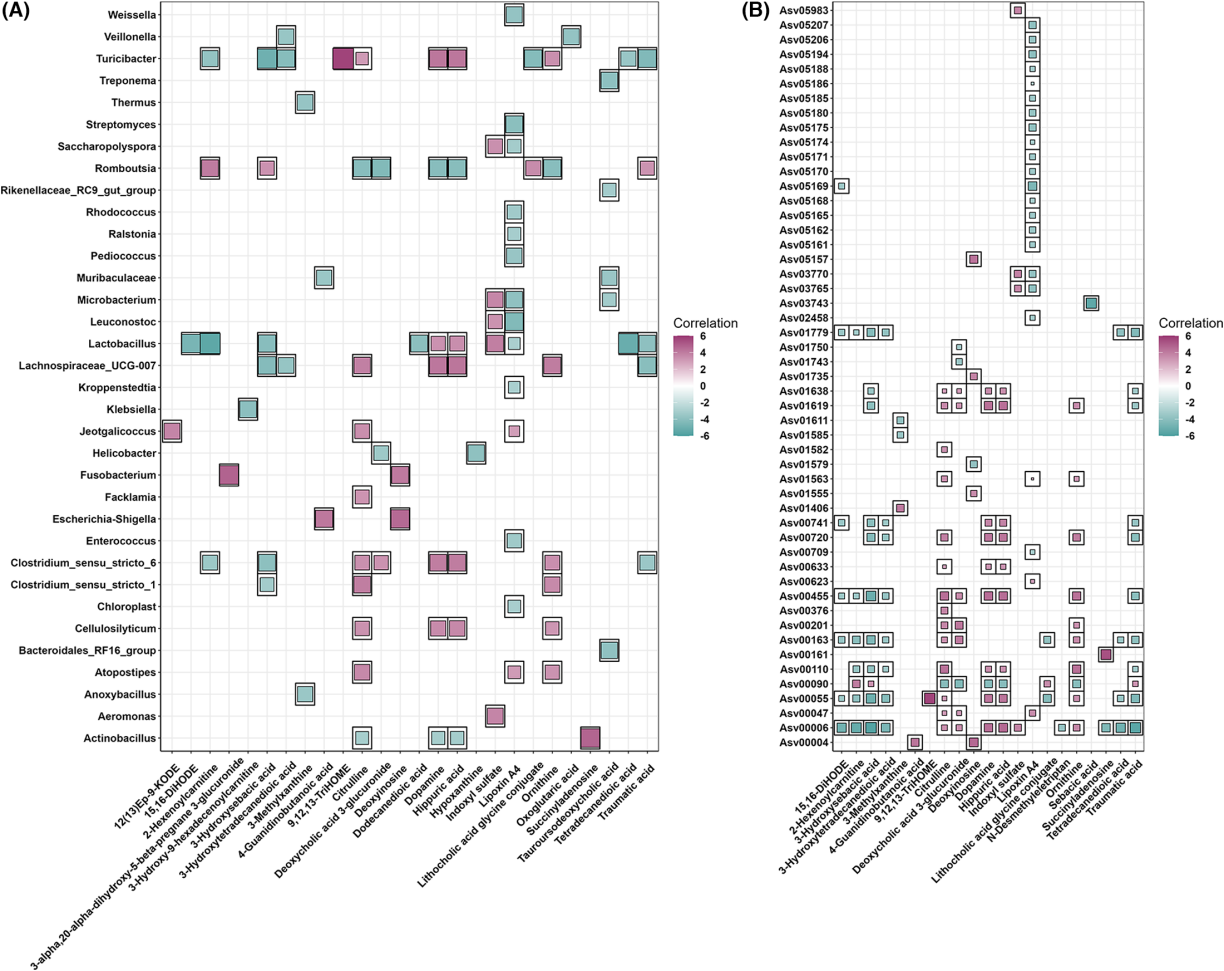
Overview of associations between serum metabolites and ileum microbiota. (A) At the genus level, the associated metabolites are coloured grey, and the associated genera are coloured successively. Each line indicates a significant correlation between a bacterium and a metabolite, with the red colour corresponding to a positive association (Z score ≥5) and the blue colour representing a negative association (Z score ≤ −5). (B) At the ASV level, the associated ASVs are coloured grey, while the associated metabolites are coloured successively. Each line indicates a significant correlation between an ASV and a metabolite, with light red corresponding to a positive association and light blue representing a negative association.

At the genus level, the association analysis by a two‐part model detected a total of 1304 associations between 584 metabolite features and 57 genera in ileum content samples at FDR < 0.05. Among these 584 metabolite features, 26 metabolites were characterized based on the Metabolomics Standards Initiative (MSI) and its classification in the HMDB database. We focused on these 26 metabolites, for which 95 significant associations related to 34 unique genera were identified. These 26 metabolites covered amino acids, organic acids, lipids, carbohydrates, and nucleotides. For instances, indoxyl sulfate was positively correlated with *Lactobacillus*, *Aeromonas*, *Microbacterium*, *Saccharopolyspora* and *Leuconosto*c (Z score ≥3.30, *p* ≤1.10 × 10^−3^). Succinyladenosine was positively correlated with *Actinobacillus* (Z score = 4.72, *p* = 3.96 × 10^−6^), while citrulline showed negative correlations with *Actinobacillus* (Z score = −3.09, *p* = 2.24 × 10^−3^). In addition, 4‐guanidinobutanoic acid was negatively associated with *Muribaculaceae* (Z score = −3.52, *p* = 4.30 × 10^−4^), but positively correlated with *Escherichia‐Shigella* (Z score = 4.13, *p* = 5.03 × 10^−5^) (Table S2; Figure 2A).

At the ASV level, we found 147 significant associations involving in 21 metabolites and 51 ASVs. 3‐hydroxysebacic acid showed the most significant and negative correlation with *Lactobacillus* (Asv00006) (Z score = −5.81, *p* = 2.05 × 10^−8^). There were ten ASVs showing negative correlations with traumatic acid, including Asv00006 (*Lactobacillus*), Asv00055 (*Turicibacter*), Asv01779 (*bacterium_RA2114*), Asv00720 (*Lachnospiraceae_UCG‐007 uncultured*), Asv00455 (*Clostridium butyricum*), Asv00163 (*Lactobacillus*), Asv00741 (*Cellulosilyticum*), Asv01619 (*Clostridium bornimense*), Asv01638 (*Clostridium bornimense*) and Asv00110 (*Clostridium_sensu_stricto_1*) (Z score ≤ −3.25, *p* ≤1.15 × 10^−3^). Furthermore, we observed that both 4‐guanidinobutanoic acid and deoxyinosine were positively associated with Asv00004 (*Escherichia‐Shigella*) (Z score = 4.21 and 4.63, *p* = 3.54 × 10^−5^ and 5.90 × 10^−6^) (Table S3; Figure 2B).

#### Associations between cecum bacteria and serum metabolite features

**FIGURE 3:**
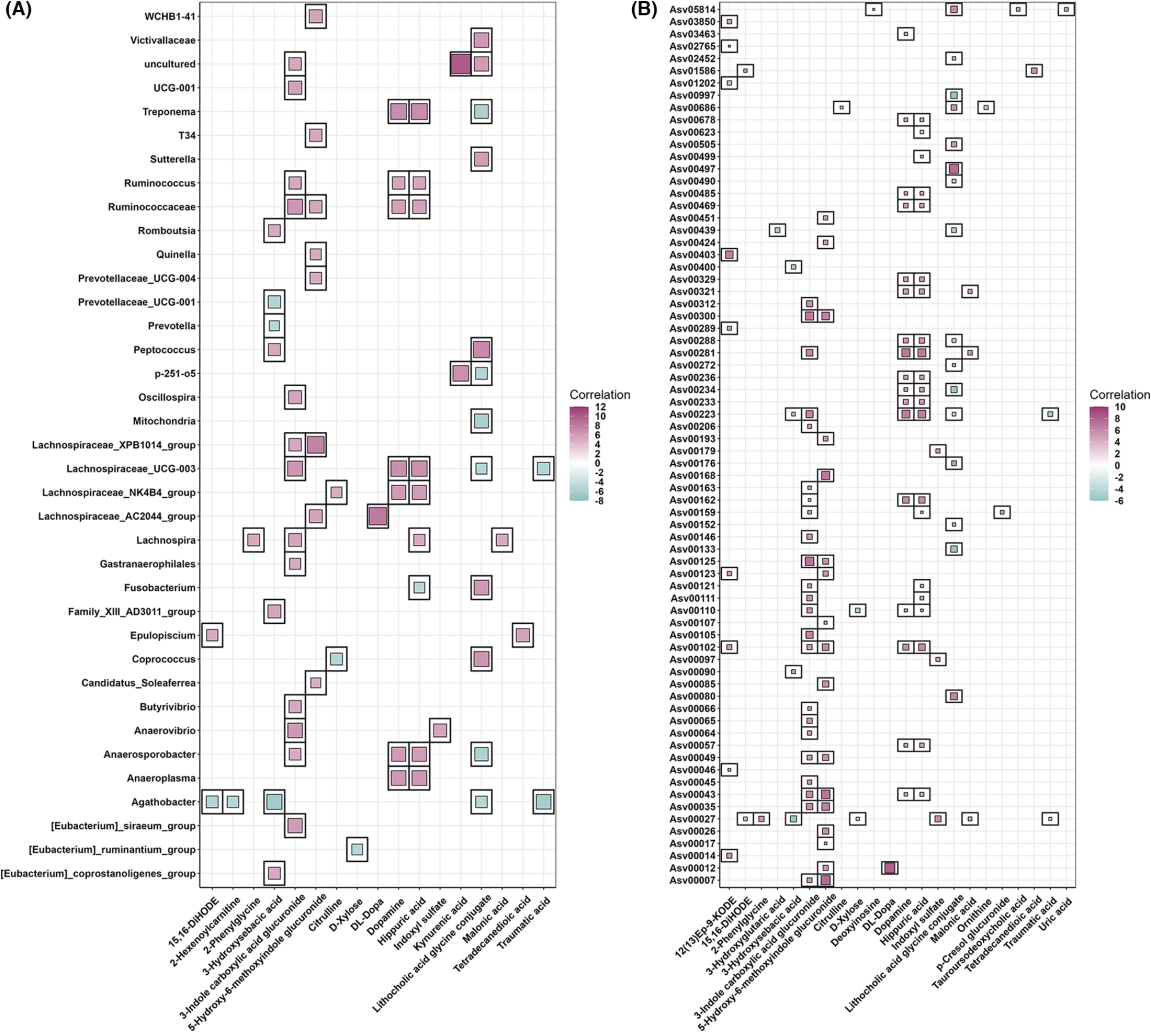
Associations between serum metabolites and cecum microbiota. (A) At the genus level, the associated metabolites are coloured grey, and the associated genera are coloured successively. Each line indicates a significant correlation between a bacterium and a metabolite, with the red colour corresponding to a positive association (Z score ≥5) and the blue colour representing a negative association (Z score ≤ −5). (B) At the ASV level, the associated ASVs are coloured grey, while the associated metabolites are coloured successively. Each line indicates a significant correlation between an ASV and a metabolite, with light red corresponding to a positive association and light blue representing a negative association.

Both microbial composition of the cecum content and serum metabolome profile were available in 300 F_6_ pigs. Potential relationships were identified between microbial compositions and serum metabolome profiles. At the genus level, we identified a total of 6400 significant associations between 725 metabolite features and 117 bacterial genera. Among them, 732 significant associations between 38 metabolites and 118 bacterial genera were retained for further discussion because these 38 metabolites could be annotated with MS/MS fragments and satisfied the annotation requirements at the Level 1 and Level 2 from the MSI. It was worth noting that the metabolites having the largest number of associations with cecum bacteria were hippuric acid (*n* = 72), followed by dopamine (*n* = 71) and lithocholic acid glycine conjugate (*n* = 66). In particular, *Lachnospiraceae_UCG‐003* had the largest number of associated metabolites (*n* = 19), followed by *Anaeroplasma* (*n* = 17), *Agathobacter* (*n* = 16), *Treponema* (*n* = 16) and *Lachnospiraceae_NK4B4_group* (*n* = 16) (Table S4, Figure 3A).

At the ASV level, a total of 1720 associations were detected, which were found between 37 metabolites and 333 ASVs. Of note, 3‐indole carboxylic acid glucuronide and indoxyl sulfate were negatively associated with the abundance of Asv00114 (*Flexispira*) (Z score = −4.10 and − 3.45, *p* = 6.01 × 10^−5^ and 6.92 × 10^−4^), but positively associated with the abundance of other 18 ASVs including Asv00097, Asv00179, and Asv00222 annotated to *Prevotella* (Z score ≥3.03, *p* ≤2.66 × 10^−3^). However, ornithine and citrulline were negatively correlated with the abundances of Asv00244 and Asv00457, which were annotated to *Prevotella*. Furthermore, the abundance of Asv00012 (*Lachnospiraceae_AC2044_group*) exhibited the most significant correlation with the DL‐dopa (Z score = 8.17, *p* = 9.04 × 10^−15^), followed by 5‐hydroxy‐6‐methoxyindole glucuronide (Z score = 5.51, *p* = 3.52 × 10^−8^). Another indole derivate, 3‐indole carboxylic acid glucuronide was positively correlated with 159 ASVs, including Asv00125 (*Anaerovibrio*), Asv00300 (*WCHB1‐41*) and Asv00105 (*Actinobacillus*) (Z score ≥6.30, *p* ≤1.07 × 10^−9^) (Table S5, Figure 3B).

#### Associations between faecal microbiota composition and serum metabolite features

**FIGURE 4:**
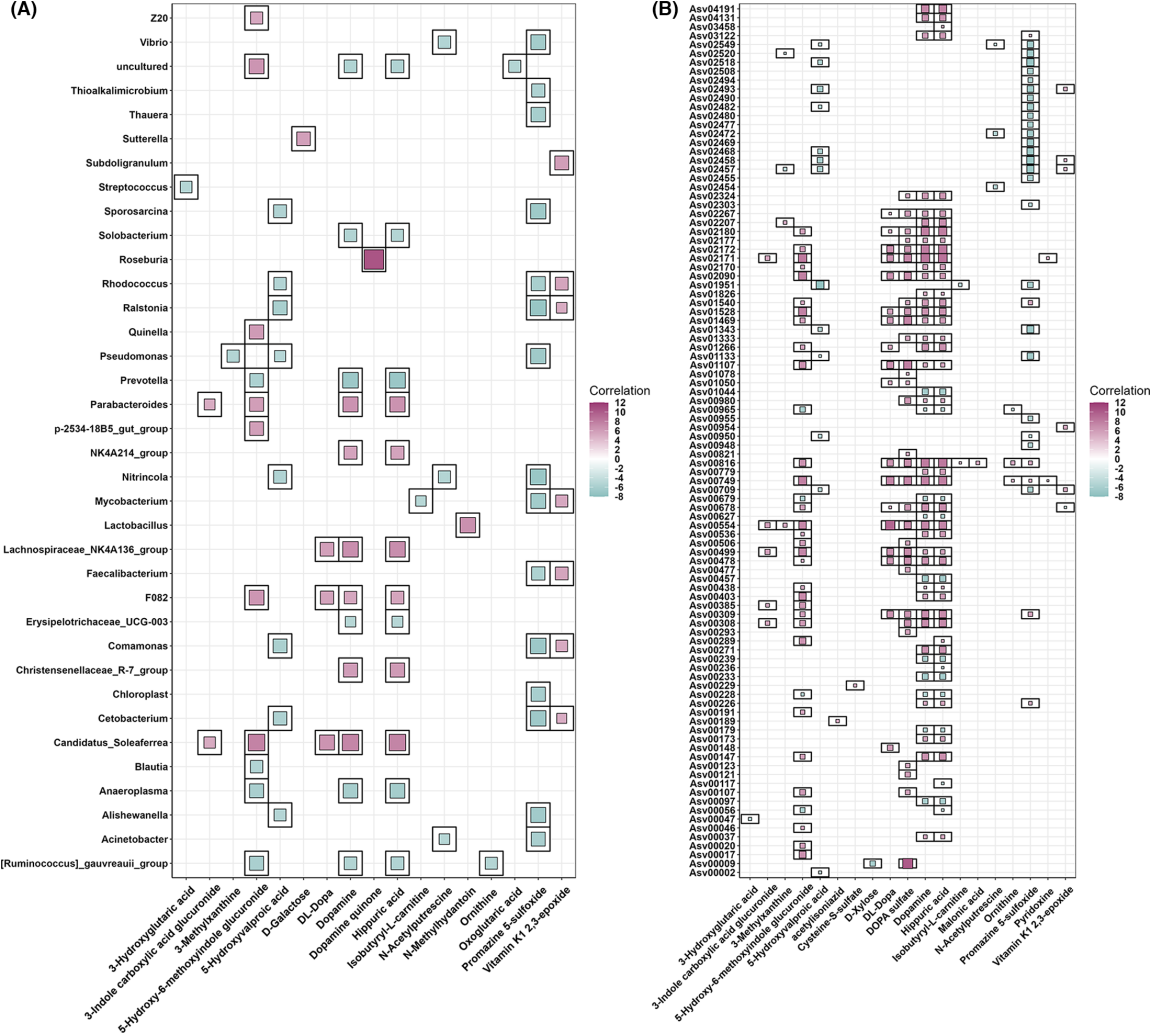
Depiction of associations between serum metabolites and faeces microbiota. (A) At the genus level, the associated metabolites are coloured grey, and the associated genera are coloured successively. Each line indicates a significant correlation between a bacterium and a metabolite, with the red colour corresponding to a positive association (Z score ≥5) and the blue colour representing a negative association (Z score ≤ −5). (B) At the ASV level, the associated ASVs are coloured grey, while the associated metabolites are coloured successively. Each line indicates a significant correlation between an ASV and a metabolite, with light red corresponding to a positive association and light blue representing a negative association.

To identify the correlations between faecal microbes and serum metabolite features, 522 pigs with both 16S rRNA gene sequencing and serum metabolome data were used for correlation analyses. At the genus level, a total of 48,561 significant correlations were identified between 2421 metabolite features and 129 genera. As described above, a total of 757 significant associations between 51 annotated metabolites and 102 bacterial genera were retained for further analyses. Notably, these 50 metabolites mainly belonged to fatty acids, amino acids, nucleotides and purine derivatives, such as 2‐hexenoylcarnitine, D‐tryptophan, ribothymidine and hippuric acid. The metabolites having the largest number of correlations with stool microbiota were hippuric acid (*n* = 70), followed by dopamine (*n* = 66), promazine 5‐sulfoxide (*n* = 47) and 5‐hydroxyvalproic acid (*n* = 45). Furthermore, the genera with the largest number of associations with metabolites were *Sutterella* (*n* = 17), *Pseudomonas* (*n* = 16), *Cetobacterium* (*n* = 15), *Comamonas* (*n* = 15), *Sporosarcina* (*n* = 15) and *Rhodococcus* (*n* = 15) (Table S6, Figure 4A).

We further identified 2252 associations between 51 metabolites and 350 ASVs. Hippuric acid showed a strongly positive correlation with Asv02171, Asv00816 and Asv00749 annotated to *Christensenellaceae_R‐7_group*, Asv02172 (*[Eubacterium]_coprostanoligenes_group*) and Asv02180 (Lachnospiraceae) (Z score ≥7.89, *p* ≤2.92 × 10^−15^), but was negatively correlated with Asv00457 (*Prevotella*), Asv00097 (*Prevotella*), and Asv00239 (*Anaeroplasma*) (Z score ≤ −5.98, *p* ≤4.41 × 10^−9^). N‐acetylputrescine was negatively with Asv02454 (*Oscillospiraceae_UCG‐005*) (Z score = −5.33, *p* = 1.01 × 10^−7^), but positively correlated with Asv00816 and Asv00749 (*Christensenellaceae_R‐7_group*) (Z score ≥3.96, *p* ≤7.62 × 10^−5^), which was similar to the association patterns of hippuric acid. The most significantly positive correlations were observed between Asv00009 (*Sphaerochaeta*) and DOPA sulfate (Z score = 10.09, *p* = 5.51 × 10^−22^). 5‐hydroxy‐6‐methoxyindole glucuronide was strongly correlated with Asv02171 and Asv00749 (*Christensenellaceae_R‐7_group*), and Asv02090 (*Clostridia_vadinBB60_group*) (Z score ≥7.72, *p* ≤6.21 × 10^−14^). However, Asv00965 (*[Ruminococcus]_gauvreauii_group*) and two ASVs belonging to *Prevotella* (Asv00056 and Asv00679) showed the strongly negative correlations with 5‐hydroxy‐6‐methoxyindole glucuronide (Z score ≤ −5.57, *p* ≤4.20 × 10^−8^) (Table S7; Figure 4B).

We further used another statistical method, the Spearman rank correlation analysis to validate these correlation results. The Spearman rank correlation analysis was performed between 128 serum metabolites that could be precisely annotated, and 75 bacterial ASVs in ileum content, 391 ASVs in cecum content and 404 ASVs in faeces samples. All these ASVs passed the quality control, had the relative abundance >0.05% and were presented in at least 5% of tested samples from each of three gut locations. The significance threshold was adjusted for the multiple tests with Benjamini‐Hochberg (BH). It was worthy to note that 94% (138 of 145) of the significant associations identified by the two‐part models were repeated by the Spearman rank correlation analysis in ileum samples. Similar results were obtained in cecum content and faeces samples, in which 88% (1523 of 1720) and 86% (1938 of 2252) of significant associations were confirmed by the Spearman rank correlation analysis (Table S8).

### Significant associations between serum metabolites and microbial taxa commonly identified in more than one gut location

#### Significant correlations commonly identified across three gut locations

Although anatomical structure, physiological characteristics and gut microbiota compositions were distinct between small and large intestines (Figure 1D), we identified 15 significant associations between five ASVs and nine metabolite features that were shared across ileum, cecum, and faeces samples (Figure S1C). Based on the HMDB database, among these nine metabolite features, only three features could be annotated and belonged to hippuric acid, dopamine and deoxycholic acid 3‐glucuronide (Table S9). These three metabolites were significantly associated with four ASVs. Both hippuric acid and dopamine were correlated with Asv00006 (*Lactobacillus*), Asv00110 (*Clostridium_sensu_stricto_1*), and Asv00055 (*Turicibacter*) in all three gut locations. The association between deoxycholic acid 3‐glucuronide and Asv00201 (*Lactobacillus*) was also simultaneously detected in all three gut locations.

#### Significant associations commonly identified across two gut locations

Considering the closer anatomical proximity of the ileum and cecum, we observed 102 metabolite features that were significantly correlated with the microbiota composition of both ileum and cecum. Here, we focused on 32 pairs of associations between 11 metabolite features that could be annotated to specific metabolites and eight ASVs (Table S10). Among them, 15,16‐DiHODE was negatively correlated with Asv00455 (*Clostridium butyricum*) (Z score = −3.39 and −3.68, *p* = 7.04 × 10^−4^ and 2.30 × 10^−4^) in both ileum and cecum contents. Both Asv00455 (*Clostridium butyricum*) and Asv00110 (*Clostridium_sensu_stricto_1*) showed a negative relationship with the 2‐hexenoylcarnitine in both gut locations (Z score ≤ −3.36, *p* ≤9.02 × 10^−4^). We also identified positive correlations of indoxyl sulfate with Asv00006 (*Lactobacillus*) in both ileum and cecum contents (Z score ≥3.79, *p* ≤1.50 × 10^−4^). Interestingly, besides Asv00006, Asv00110 and Asv00455, which were associated with hippuric acid and dopamine in all three gut locations as described above, we further identified Asv00455 (*Clostridium butyricum*) and Asv00090 (*Romboutsia*) that were associated with hippuric acid and dopamine in both ileum and cecum contents (Z score ≥3.83 and ≤ −4.14, *p* ≤1.55 × 10^−4^).

A larger number of associations was found to be shared between cecum content and faeces samples (1324 associations) (Figure S1C). Among these associations, 251 pairs of associations were found between ten metabolites and 109 ASVs. Most of the associations shared between cecum and faeces samples were identified at hippuric acid (with 132 ASVs), followed by dopamine (with 126 ASVs) and 5‐Hydroxy‐6‐methoxyindole glucuronide (with 110 ASVs). We also found 26 ASVs that were correlated with 3‐indole carboxylic acid glucuronide. Malonic acid was positively associated with Asv00037 (*Bacteroides*), Asv00107 (*Quinella*), and Asv00499 (*Clostridia_vadinBB60_group*) in both cecum content and faeces (Z score ≥3.31, *p* ≤1.49 × 10^−3^). It is worth noting that malonic acid was positively correlated with Asv00097 (*Prevotella*), Asv00238 (Lachnospiraceae), Asv00233 (*Erysipelatoclostridiaceae_UCG‐004*), Asv00236 (*Anaeroplasma*) and Asv00239 (*Anaeroplasma*) in cecum content samples (Z score ≥3.19, *p* ≤1.12 × 10^−3^), but showed a negative correlation in faeces samples (Z score ≤ −3.24, *p* ≤1.28 × 10^−3^) (Table S11).

### The proportion of the variance of serum metabolites explained by the gut microbiota

The percentages of serum metabolite variance that could be explained by the microbiota of three gut locations were estimated. Overall, the microbiota in the cecum content could explain more variance than that in the other gut locations. We found deoxycholic acid 3‐glucuronide, 3‐methylxanthine, and dopamine, whose variance in serum samples explained by the ileum microbiota (ranging from 2.21% to 6.24%) was higher than that of other metabolites at a cutoff *p* value of 0.05 (Figure S2A–C; Table S12). In particular, the variance of deoxycholic acid 3‐glucuronide was mostly contributed by the intestinal microbiota (6.24%). Based on the cecum microbiota, the explanation of the variance serum metabolites by microbiota ranged from 0.54% to 8.42% (Table S13). The top three percentages of the variations explained by the cecum microbiota were identified for the metabolites of indoxyl sulfate (8.42%), malonic acid (5.61%) and 2‐phenylglycine (4.97%) (Figure S2D–F). Based on faecal microbiota, the variance of serum metabolites explained by the faecal microbiota varied from 0.32% to 6.39% (Table S14). The largest percentage of the variations explained by the faecal microbiota was estimated for D‐galactose (6.39%), 3‐hydroxy‐cis‐5‐tetradecenoylcarnitine (4.79%) and oxoglutaric acid (4.00%) (Figure S2G–I).

### Microbiota‐related metabolites showing significant associations with fat deposition

We identified a close relationship between porcine fatness traits and gut bacteria in our previous studies (Chen et al., 2021; He et al., 2016). In the present study, hundreds of significant associations were detected by computing the correlations between serum metabolite features and gut microbiota from three gut locations. Notably, from the correlation results in this study, a number of metabolites that have been reported to be related to fat deposition were found to be associated with gut microbiota. Hence, to verify the potential role of serum metabolites in the process of host fat deposition, we quantified the abundances of 108 metabolites (See methods) using targeted metabolome measurements in serum samples from 30 experimental pigs which were selected from the studied population due to their extreme phenotypic values of backfat thickness, including 15 pigs with high backfat thickness (high‐fatness group, HFG) and 15 individuals with low backfat thickness (low‐fatness group, LFG) (HFG vs. LFG, *p* = 3.38 × 10^−6^).

**FIGURE 5:**
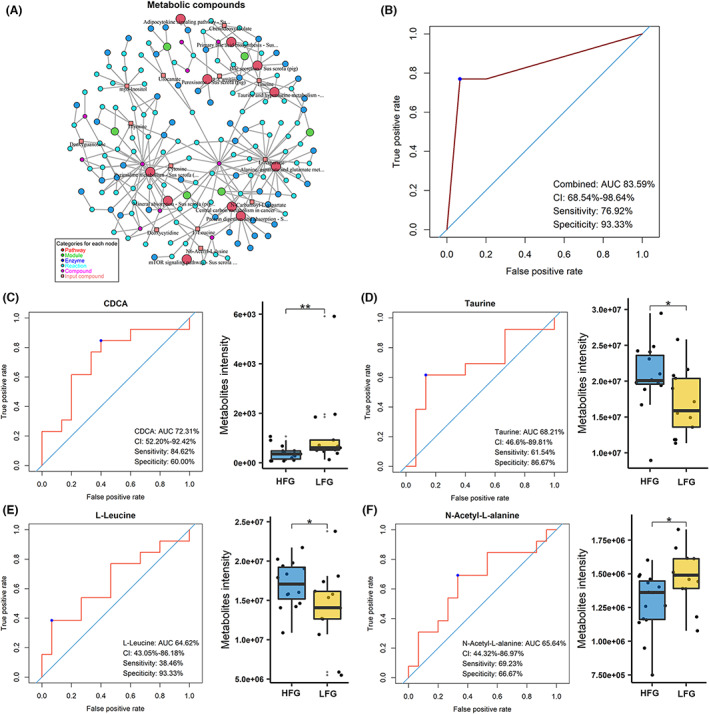
KEGG pathways that 19 differential metabolites involved in, and metabolite biomarkers selected for predicting host fat deposition. (A) The pathways that 19 differential metabolites involved in. The pathway network was constructed based on the sub‐database of KEGG pathways related to the pig. The 19 differential metabolites were matched to the sub‐database of KEGG pathways. Intermediates associated with differential metabolites, including enzymes, reactions, compounds, etc. were also shown in the network. (B) Prediction of disparate fatness traits using four metabolites with a higher prediction performance of 82.56%. (C‐F) The receiver operating characteristic curve that was used to evaluate the prediction performance of backfat thickness for each of the four metabolites.

PLS‐DA analysis revealed that the serum metabolite compositions of the HFG group were significantly different from that of the LFG group (Figure S3A). A total of 19 metabolites with VIP >1.0 and *p* < 0.05 were identified as differential serum metabolites between HFG and LFG groups (Table 1). These 19 metabolites were implicated in eleven pathways including mTOR signalling pathway, Protein digestion and absorption pathway, ABC transporters, Primary bile acid biosynthesis, and Taurine and hypo‐taurine metabolism (Figure 5A). Based on these 19 metabolites, an optimal model was constructed by binary logistic regression and the stepwise selection method. We identified that a combination of four metabolites including CDCA, taurine, L‐leucine and N‐acetyl‐L‐alanine could be used as the metabolite biomarker panel which could discriminate between pigs with high backfat thickness. L‐leucine and taurine were associated with increased backfat thickness, while N‐acetyl‐L‐alanine and CDCA were associated with decreased backfat thickness (Figure S4). We found that the diagnostic performance by using all four metabolites was notably better than that using only one of four metabolites, with AUC values of 83.59% versus 72.31%, 68.21%, 64.62% and 65.64% (Figure 5B–F). These findings implied that fatness‐related metabolite biomarkers could be used as potential markers for porcine fat deposition.

**TABLE 1:** The differential metabolites between pigs with high and low backfat thickness at the criteria of *p* <0.05 and VIP >1.

Metabolite	HFG (mean ± sd)	LFG (mean ± sd)	*p*‐value	VIP‐value
Deoxycytidine	7524650.47 ± 1787960.83	10261661.86 ± 3644838.95	0.003	1.856
N4‐acetylcytidine	498148.83 ± 129393.8	885812.44 ± 367940.1	0.006	1.824
N6‐acetyl‐L‐lysine	1252582.78 ± 303840.54	1618825.37 ± 347733.35	0.006	1.753
CDCA	391.71 ± 288.81	1221.82 ± 1431.30	0.007	1.213
Thymine	286911.89 ± 141311.15	444600.68 ± 160149.87	0.015	1.379
N‐carbamoyl‐L‐aspartic acid	874398.2 ± 257244.08	1107077.74 ± 284038.44	0.017	1.409
Urocanic acid	511432.2 ± 264806.63	982100.55 ± 566281.86	0.019	1.618
N‐acetyl‐L‐alanine	1285865.24 ± 214722.31	1520824.39 ± 269371.19	0.022	1.458
DL‐2‐aminooctanoic acid	284664.2 ± 138429.51	250261.51 ± 283405.95	0.022	1.705
L‐aspartic acid	810065.53 ± 222873.28	1023067.74 ± 295513.25	0.029	1.553
HDCA	1454.01 ± 1059.12	3895.91 ± 4720.44	0.033	1.089
Propionylglycine	149555.08 ± 128593.45	342952.66 ± 264718.68	0.033	1.408
Deoxyguanosine	95483.53 ± 34617.71	159989.77 ± 78466.73	0.037	1.49
Cytosine	185839.6 ± 52617.98	319837.35 ± 257943.41	0.037	1.498
L‐carnitine	112963514.6 ± 31433215.33	90611112.4 ± 20540326.78	0.041	1.485
L‐leucine	16996899.4 ± 2686845.46	16897518.4 ± 11843436.82	0.041	1.615
r‐MCA	422.21 ± 304.26	883.53 ± 728.51	0.046	1.38
myo‐inositol	2368862.09 ± 573876.73	2855503.51 ± 859042.1	0.046	1.188
Taurine	20702685.18 ± 4336622.99	18854459.04 ± 7126356.53	0.046	1.509

Abbreviations: HFG, High fatness group; LFG, Low fatness group.

**FIGURE 6:**
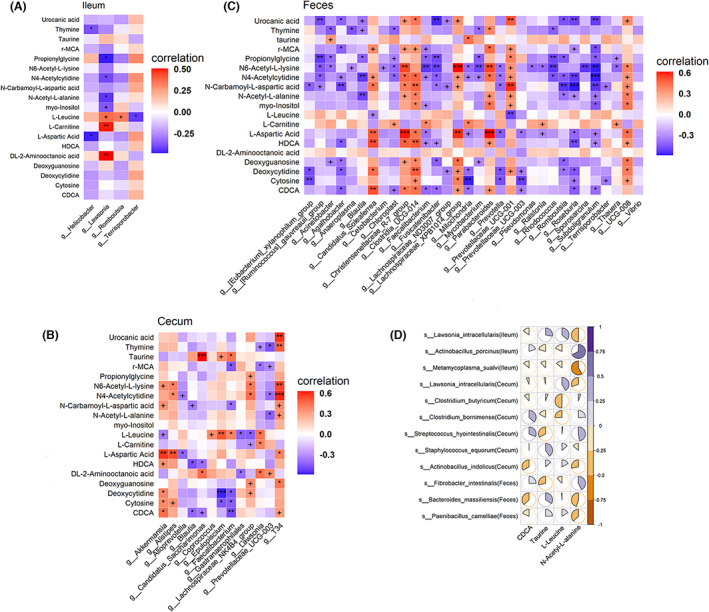
Serum metabolite associated with porcine fat deposition and its correlations with fatness‐associated bacteria. (A‐C) Heatmaps of Spearman's rank correlation coefficient between 19 differential metabolites and fatness‐associated genera in the ileum, cecum, and faeces samples. (D) Pie charts of correlations between four metabolites chosen for predicting backfat thickness and bacterial species associated with backfat thickness in three locations. It shows the associations between metabolic biomarkers and the differential gut bacteria derived from a comparison between high fatness thickness (HFG) and low fatness thickness (LFG) groups in the ileum, cecum, and faeces samples. +, *, **, and *** mean significance levels of *p* < 0.1, *p* < 0.05, *p* < 0.01, *p* < 0.001 between HFG and LFG groups.

We then analysed the correlations between 19 fatness‐associated metabolites and gut microbiota. At the genus level, more faecal and cecum bacteria were found to be significantly correlated with these 19 serum metabolites than that of ileum bacteria. In more detail, we identified four, 13 and 32 genera showing significantly different abundances between HFG and LFG groups in ileum, cecum, and faeces samples. The correlation analyses between these fatness‐associated genera and 19 fatness‐associated metabolites identified multiple significant associations for each genus (Figure 6A–C). For example, *Lawsonia*, *Helicobacter* and *Romboutsia* were enriched in the ileum of HFG pigs. These genera were positively and significantly correlated with DL‐2‐aminooctanoic acid and L‐leucine, but negatively associated with N4‐acetylcytidine, N6‐acetyl‐L‐lysine, N‐carbamoyl‐L‐aspartic acid, N‐acetyl‐L‐alanine, urocanic acid, deoxycytidine, thymine and CDCA (Figure 6A). *Akkermansia* was enriched in the cecum of LFG pigs, and it was positively associated with L‐aspartic acid, cytosine, deoxycytidine, thymine, N6‐acetyl‐L‐lysine, N‐carbamoyl‐L‐aspartic acid, CDCA and HDCA (Figure 6B). *Christensenellaceae_R‐7_group* showed the same correlation patterns displayed by *Akkermansia*. It had a higher abundance in the faeces of the LFG group, and was positively associated with L‐aspartic acid, N4‐acetylcytidine, N6‐acetyl‐L‐lysine, N‐carbamoyl‐L‐aspartic acid, N‐acetyl‐L‐alanine, urocanic acid, deoxyguanosine, γ‐MCA, HDCA, and CDCA. However, *Prevotella* had a higher abundance in the faeces of HFG pigs than that in the faeces of LFG pigs. It was positively associated with L‐aspartic acid, N4‐acetylcytidine, N6‐acetyl‐L‐lysine, N‐carbamoyl‐L‐aspartic acid, N‐acetyl‐L‐alanine, deoxyguanosine, cytosine and CDCA (Figure 6C). Then, we focused on four metabolite biomarkers identified above, and analysed their associations with differential bacterial species between HFG and LFG groups. As expected, *Actinobacillus porcinus* in the ileum showed positive correlations with CDCA and N‐acetyl‐L‐alanine, but was negatively associated with L‐leucine and taurine. *Lawsonia intracellularis* were positively associated with L‐leucine, but negatively associated with N‐acetyl‐L‐alanine and CDCA. In cecum content samples, *Clostridium butyricum* was positively associated with N‐acetyl‐L‐alanine and taurine, but negatively correlated with CDCA and L‐leucine. In faeces samples, *Bacteroides massiliensis* and *Paenibacillus camelliae* were negatively associated with CDCA and N‐acetyl‐L‐alanine, but positively correlated with taurine and L‐leucine (Figure 6D).

## DISCUSSION

This work identified hundreds of significant associations between gut bacteria and serum metabolites by using gut microbial composition data from three different gut locations. Notably, many microbiota‐associated metabolite features were consistent with those identified previously. However, this study also found some microbiota‐associated metabolites that, to our knowledge, have not been reported previously. Furthermore, some correlations between serum metabolites and gut microbiota depended on gut locations. More predominantly, we observed that hippuric acid, dopamine and deoxycholic acid 3‐glucuronide were correlated with multiple bacterial taxa independent of gut locations, which meant extensive relationships between these metabolites and gut bacteria. To our knowledge, this was the first report describing the associations of serum metabolite profiles with gut microbiota in a large‐scale of samples from different gut locations. All experimental pigs were fed with the same diets under unified indoor conditions, and all samples were collected at the same age when pigs were slaughtered. Thus, these eliminated the influences of diets, environmental factors, and host age on the compositions of gut microbiota and serum metabolome, and facilitated to evaluate the effect of gut microbiota on serum metabolome profiles.

Untargeted metabolome measurement was characterized by its broad spectrum in quantifying as many as 5366 uncharacterized metabolites. However, we were only able to assign metabolite features to only 128 compounds. The reason for the limited annotations was mainly due to the lack of complete and sophisticated databases. As byproducts of metabolic processes, the increment or diminishment of the concentrations of metabolites is linked to the modulation of systemic metabolism, which in turn alters phenotype within certain genetics‐bound limits (Guijas et al., 2018). Hence, we were only concerned with those metabolites with an assigned and reliable compound name in the HMDB database according to MS/MS fragments and satisfied the requirements of Level 1 and Level 2 from the MSI.

Both positive and negative associations were observed between microbial taxa and serum metabolites. It is easily explained by (i) the metabolites might act as preferred substrates to promote microbial growth or a detrimental substrate that inhibits the growth of microorganisms; (ii) Some metabolites could shape the microbial composition by regulating the ecological niche of synergetic or antagonistic bacteria (Franzosa et al., 2019); (iii) bacteria could indirectly affect serum metabolites mediating by a third party. For example, host genetics should regulate the abundances of serum metabolites and then influence gut microbiota, or host genetics could also regulate the abundances of gut microbiota and then influence serum metabolites, or gut microbiota affects host gene expression, and then influences serum metabolites.

Although hundreds of gut microbiota‐metabolite associations were identified, the largest percentage of serum metabolite variance explained by the gut microbiota was only 8.42%. This percentage was similar to the range reported in a previous report (Fu et al., 2015). We speculated that the reason might be due to the complexity of influencing factors, such as environments, host genetics, age and physiological status.

Previous reports suggested that Lipoxin A4, an endogenous eicosanoid of arachidonate metabolism, can actively promote the resolution of inflammation (McMahon et al., 2001) and increase antibiotic efficacy (Thornton et al., 2021). In this study, it was correlated with the largest number of ASVs in the ileum, and the majority of bacteria showed negative correlations with lipoxin A4 (20 ASVs), indicating a potential role of lipoxin A4 in inhibiting the growth of gut bacteria. Hippuric acid has been reported to be synthesized by the conjugation of bacteria‐produced benzoic acid with glycine and modulated by the intestinal microbiome (Wikoff et al., 2009). In addition, hippuric acid showed a negative correlation with visceral fat weight and obesity (Burmester et al., 2007; Calvani et al., 2010). In this study, there were 202 ASVs in the cecum and 215 ASVs in the faeces showing significant associations with hippuric acid. Consistent with the previous report (Sieber et al., 1995) which found that hippuric acid can consequently be converted to benzoic acid by lactic acid bacteria, the abundance of hippuric acid was significantly associated with *Lactobacillus*, *Clostridium_sensu_stricto_1* and *Turicibacter* in all three gut locations. Combining the result from this study and the association of hippuric acid with fatness (Burmester et al., 2007, Calvani et al., 2010), we suggested that *Lactobacillus* may mediate host fat accumulation through hippuric acid.

Several reports revealed that dopamine is a key modulator of learning and motivation (Berke, 2018; Liu, Chong, et al., 2020; Liu, Xie, et al., 2020). We showed that dopamine was tightly correlated with Asv00006 (*Lactobacillus*), Asv00055 (*Turicibacter*) and Asv00110 (*Clostridium_sensu_stricto_1*) across three gut locations. These results were consistent with the reports that serum dopamine levels were altered obviously in GF mice which were treated with *Lactobacillus* (Galland, 2014; Liu, Chong, et al., 2020; Liu, Xie, et al., 2020) and in pigs which were fed with *Clostridium butyricum*‐based compound probiotic (Cao et al., 2019). Deoxycholic acid 3‐glucuronide is a metabolite of deoxycholic acid generated in the liver. It participates in the emulsification of lipids for absorption in the intestine and contributes to intestinal tumours (Sun et al., 2018). We found that it was significantly correlated with Asv00201 (*Lactobacillus*) in all three gut locations. Further investigations would warrant to confirm the function roles of *Lactobacillus* in lipid absorption and intestinal diseases through deoxycholic acid 3‐glucuronide or by interacting with deoxycholic acid 3‐glucuronide.

The number of significant correlations between gut bacteria and serum metabolites quantified by targeted metabolome analysis was low. We proposed that this might be due to (i) the smaller sample size in targeted metabolome analysis (28 samples) with limited bacterial taxa, (ii) 21 out of 108 targeted metabolites belong to bile acid metabolism‐related metabolites which were enriched in ileum samples, but not in cecum and faeces samples. The lower number of metabolites measured and the lower number of genera and ASVs resulted by a small sample size led to fewer significant associations. Interestingly, many metabolites measured by targeted metabolome, such as ornithine and citrulline, were correlated with a range of different bacteria in three gut locations. More specifically, *Lachnospiraceae_UCG‐007*, *Lactobacillus* and *Clostridium butyricum* were the main bacterial taxa that showed positive associations with both ornithine and citrulline in ileum samples, and *Clostridium butyricum* was also positively associated with these metabolites in the cecum. However, these associations were not detected in stool samples. These different correlation results across gut locations might be caused by different microecology systems in which different interaction networks of gut microbiota existed or by different physiological functions of gut locations. This result suggested that gut microbial compositions in different gut locations would be taken into account when we consider regulating porcine fat deposition through modulating the gut microbiota composition in the future.

We found that a metabolite panel consisting of CDCA, taurine, L‐leucine and N‐acetyl‐L‐alanine could be used to predict host fat deposition with high accuracy. Clinical tests of serum metabolites have been a considered reliable, but expensive method, compared with fast, non‐invasive, and relatively inexpensive ultrasound. However, the prediction of potential fat deposition using these four serum metabolite biomarkers in growing pigs should be a promising strategy to reduce the fat deposition of pigs, which should improve feed efficiency and increase the profits of the pig industry. It is necessary to mention that these biomarkers were selected from the measurement at slaughter. A longitudinal study would be necessary to further confirm this result. More importantly, this finding provided an important cue that these four metabolite biomarkers might be used to predict human obesity because pigs have been considered as an important biomedical model for studying human obesity (Lunney et al., 2021; Renner et al., 2020). As a typical primary bile acid produced by the host, CDCA is easily decomposed by bacteria in the intestinal tract. We found significantly decreased serum CDCA level and increased taurine level in the HFG group compared with that in the LFG group. This result agreed with previous reports (Pineda Torra et al., 2003; Shapiro et al., 2018). CDCA promotes the release of glucagon‐like peptide‐1 in diabetic patients, likely by activating GPBAR1. BAs, including CDCA, are involved in the digestive process, maintaining intestinal microbial composition, balancing lipid and glucose metabolism, insulin sensitivity, and innate immunity (Chen et al., 2020; Hofmann, 2009; Song, Cai, et al., 2019; Song, Zhong, et al., 2019; Wahlstrom et al., 2016). CDCA binding to the nuclear hormone receptor farnesoid X receptor leads to increased energy expenditure, inflammation and the configuration of gut microbiomes, which might impact fat deposition in the liver and even peripheral tissue. Taurine is a conditionally essential amino acid, as well as an antioxidant, which is necessary for the absorption of lipids from the digestive tract. A previous work showed that oral gavage of taurine can ameliorate obesity and insulin resistance by modulating the inflammatory process (Wen et al., 2019). However, it was strange that taurine had the higher abundance in HFG pigs, and was positively correlated with *Candidatus Saccharimonas* that was also enriched in obese pigs in cecum content samples. This suggested the complex mechanism of the interaction between taurine and gut microbiota in affecting host fat deposition. Consistently, *Candidatus Saccharimonas* had a higher abundance in obese mice (Song, Cai, et al., 2019; Song, Zhong, et al., 2019). Moreover, the negative correlation between taurine and *Clostridium bornimense* in ileum content samples further confirmed the tight interaction between gut microbiome and serum BA level. This also highlighted the roles of microbial activity in modulating the metabolism of bile acids, and in further regulation of host fat deposition.

Branched chain amino acid (BCAA) is involved in host inflammation, insulin resistance, and obesity in humans (Pedersen et al., 2016). We noted that the abundance of *Clostridium Butyricum* was higher in the LFG group. A previous report stated that *Clostridium Butyricum* is able to convert L‐leucine (Butel et al., 1995). *Clostridium Butyricum* has been considered a probiotic that can protect intestinal barrier function and decrease intestinal mucosal permeability in mice and humans. Increased evidences have suggested that excessive accumulation of BCAAs could lead to hyperactivation of the mTOR signalling pathway, induction of oxidative stress, insulin resistance, and/or impaired glucose metabolism (Wolfson et al., 2016). The protective effects of *Clostridium Butyricum* on the intestinal barrier function could be mediated by the Akt/mTOR signalling pathway (Liu, Chong, et al., 2020; Liu, Xie, et al., 2020). In this study, the pathway analysis also identified the enrichment of differential metabolites in the mTOR signalling pathway (Figure 5A). Both the abundance of *Prevotella* and the level of serum L‐leucine were increased in pigs with higher fat deposition. In our previous study, we reported positive relationships between 13 bacterial species belonging to the *Prevotella* genus in the gut and pig fat deposition using metagenomic sequencing data, and particularly found that *Prevotella copri* increased the concentration of BCAA in serum, which causes host chronic inflammatory response through the mTOR signalling pathway (Chen et al., 2021). Besides, *Prevotella copri* manifested positive or negative associations with host inflammation, insulin resistance and obesity in humans and mice depending on diets (De Vadder et al., 2016; Pedersen et al., 2016). Just like that reported in previous studies (Leyrolle et al., 2021; You et al., 2022), we found the enrichment of *Coprococcus* and the depletion of *Akkermansia* in the HFG group. In addition, *Coprococcus* showed a positive correlation with L‐leucine, while *Akkermansia* revealed an obvious negative correlation with L‐leucine. *Lawsonia intracellulari*, commonly associated with diarrhoea, is the causative agent of ileitis and an obligate intracellular bacterium in pigs. The elevated abundance of pathogenic bacteria including *Lawsonia intracellularis*, *Metamycoplasma sualvi* and *Actinobacillus indolicus* were noticed in the gut of HFG pigs, and were negatively correlated with CDCA and N‐acetyl‐L‐alanine. *Lawsonia intracellularis* and *Actinobacillus indolicus* were positively associated with taurine and L‐leucine which were also enriched in HFG pigs. These bacterial species probably triggered host chronic inflammatory responses, and resulted in excessive fat accumulation. These results suggested a possible role of these bacteria and their related metabolites in pig fat deposition.

In conclusion, we identified a myriad of associations between serum metabolite features and gut microbiota from three gut locations. Most of these associations are specific to each gut location. However, there were 16 metabolites that were identified to be associated with the microbiota across either two or three gut locations. These results might suggest that metabolic potentials and derived metabolites of gut bacteria depended on the enteric environments or microbial compositions. A metabolite panel consisting of CDCA, taurine, L‐leucine and N‐acetyl‐L‐alanine can be used as an effective biomarker for predicting pig excessive fat accumulation. Meanwhile, L‐leucine enriched in the HFG was positively correlated with the abundance of *Prevotella* in faeces samples. In addition, we further confirmed the pivotal roles of gut *Prevotella*, *Clostridium Butyricum* and BCAAs in host fat accumulation. Our results gave expanded insights into the correlations between serum metabolites and microbiota in distinct gut compartments, and provided extra evidence that fat accumulation in pigs could be regulated by modulating gut microbiota and/or microbiota‐derived metabolites. However, based on the current metabolite databases, a large number of metabolite features could not be annotated to specific metabolites. The relationships between serum metabolites and gut microbiota were just obtained based on the correlation analyses. The causality would need to be further confirmed in future studies.

## EXPERIMENTAL PROCEDURES

### Animals, phenotyping and sample collection

A total of 544 pigs from the 6th generation (F_6_) of a heterogeneous intercross were involved in the current study. The heterogeneous stock was produced by crossing eight pig breeds including four Western commercial pig breeds (Duroc, Landrace, Large White and Pietrain) and four Chinese indigenous breeds (Erhualian, Laiwu, Bamaxiang and Tibetan). The F_6_ generation was generated from reciprocal mating involving in combinations of the above eight founder breeds. All experimental pigs were raised under the same indoor conditions in farm houses at Jiangxi Agricultural University (Nanchang, China). All animals were fed ad libitum with a diet containing 16% crude protein, 3100 kJ digestible energy, 0.78% lysine, 0.6% calcium and 0.5% phosphorus. All 544 F_6_ pigs, including 301 females and 243 males, were slaughtered at 247.56 (±4.54) days of age in 23 batches at a standard commercial slaughterhouse in Nanchang City. Pigs were fasted overnight (12 h) but accessed to water freely. The backfat thickness at the 3rd‐4th rib region over the left side of carcass was measured for 30 experimental pigs with the vernier calliper. Faecal samples from the rectum and luminal contents of the cecum and ileum were collected within 30 min after slaughter. A total of 522 faeces (290 female, 232 male), 300 cecum content (166 female, 134 male) and 248 ileum content (140 female, 108 male) samples were harvested and immediately dipped into liquid nitrogen. All samples were then stored in a −80°C refrigerator until use after being transported to the laboratory. Blood samples (301 females and 243 males) were collected from the porcine carotid artery into sterile centrifuge tubes when slaughtered. After leaving at room temperature for 2 h, coagulated blood vessels were centrifuged for 15 min at 3000 rpm at 4°C to separate the serum. Serum samples were stored immediately at −80°C until measurement.

### DNA extraction and 16S rRNA gene amplicon sequencing

Total microbial DNA from ileum, cecum and faecal samples was extracted using QIAamp DNA Stool Mini Kit (Qiagen, Germany) according to the manufacturer's instructions. The concentration and integrity of DNA samples were measured with a Nanodrop‐1000 (Thermofisher scientific, USA) and 0.8% agarose gel electrophoresis. The V3‐V4 hypervariable region of the 16S rRNA gene was selected and amplified with the fusion primers 338F [5′‐ACTCCTACGGGAGGCAGCAG‐3′] and 806R [5′‐GGACTACHVGGGTWTCTAAT‐3′] under a melting temperature of 56°C for 28 cycles. Libraries of amplicons were constructed and sequenced on an Illumina MiSeq platform (Illumina, USA). All 2 × 300 bp paired‐end sequencing reads from clean data sets were assembled into tags using FLASH (v.1.2.11). To avoid any statistical bias caused by uneven sequencing depth, the number of clean tags for each sample was rarefied to 19,000 (the least number of tags in tested samples) in all experimental pigs.

The further bioinformatics analyses of clean tags were performed with QIIME2 pipeline (v2022.2) (Bolyen et al., 2019) by Deblur plugins (Amir et al., 2017). Deblur denoised sequences were divided into ASVs at the 99% sequence identity. Then, the Naive Bayes classifier and classify‐sklearn algorithm in q2‐feature classifier command were used to align 16S rRNA gene sequences against the SILVA reference database (v138.1) (Quast et al., 2013) to obtain bacterial taxonomic information of each ASV. The α‐diversity indices of Shannon and observed features, the β‐diversity of gut microbiota based on Bray‐Curtis distances, and the analysis of similarity (ANOSIM) were conducted in QIIME2 (v2022.2). ASVs with low abundance (<0.05%) (Pedersen et al., 2016) were filtered from further correlation analyses.

### Untargeted metabolome profiling of serum samples

#### Sample preparation

All chemicals and reagents were of HPLC grade. Serum metabolome profiles were determined using an ultrahigh‐performance liquid chromatography‐quadrupole time‐of‐flight mass spectrometry (UPLC‐QTOFMS) (Waters, USA). A total of 544 serum samples were measured under both negative and positive ionization electrospray models. In brief, 3 mL of precooled methanol (Merck Corp) was added to 100 μL of each serum sample to extract the metabolites according to the previous report (Liu et al., 2017). The mixture was vortexed for 1 min. After incubated at −20°C in a refrigerator for 3 h, the mixture was centrifuged at 15,000 rpm for 15 min at 4°C to precipitate the proteins. And then, 200 μL of the supernatant was processed in a SpeedVac overnight. The dried metabolites were resuspended in 150 μL of water/methanol (85:15, v/v), and the extraction was placed into a sampling vial pending UPLC‐QTOFMS.

#### UPLC‐MS measurements

A sample pooled from all tested samples was treated as the quality control (QC) sample. Metabolites in tested samples were separated on a UHPLC BEH C18 column (2.1 × 100 × 1.7 μm) (Waters, USA) at 40°C with a flow rate of 0.3 mL/min. The linear gradient of column elution was set as follows: 1%–20% B at 0–3 min, 20%–50% B at 3–5 min, 50%–70% B at 5–10 min, 70%–85% B at 10–15 min, and 85%–100% B at 15–17 min, followed by a re‐equilibration step of 5 min. For electrospray positive ion mode (ES+) analysis, the solvent system was water with 0.1% formic acid (A) and acetonitrile (B). For negative ion mode (ES‐) analysis, eluent A was formed by water, and acetonitrile was used as eluent B. All samples were kept at 8°C during detection. An electrospray ionization source was used in both positive and negative modes of the mass spectrometric data. The source temperature was set at 120°C with a cone gas flow of 50 L/h, and the desolvation gas temperature was set at 350°C with a desolvation gas flow of 650 L/h. In the cases of positive and negative ion modes, the capillary voltage was set at 3.0 and 2.5 kV, and the cone voltage was at 40 V. Centroid data were collected from 50 to 1200 m/z with a scan time of 0.3 s and an interscan delay of 0.02 s over a 26 or 18 min analysis time. Leucine enkephalin was used as the lock mass (m/z 556.2771 in ES+ and 554.2615 in ES−) at a concentration of 100 ng/mL and a flow rate of 5 μL/min for all analyses. A total of 1 μL extraction of each sample was used for measurement. The pooled QC sample was inserted per twelve tested samples to monitor system stability.

#### Generation of serum metabolome dataset

Data acquisition and system control were performed by MassLynx software (Waters, USA). MetaScope in Progenesis QI software (Nonlinear Dynamics; Rusilowicz et al., 2016) was used to annotate the compounds of metabolite features not only based on neutral mass, isotope distribution and retention time, but also based on collisional cross‐sectional area and MS/MS fragmentation data in the Human Metabolome Database (HMDB) (http://www.hmdb.ca) with a mass error of 10 ppm or less. We obtained the ion intensity of each peak and generated a three‐dimensional matrix containing arbitrarily assigned peak indices (retention time‐m/z pairs), ion intensities (variables), and sample names (observations). The raw matrix was further filtered by removing peaks with missing values (ion intensity = 0) in more than 80% of tested samples and 50% of the QC samples. Each retained peak was then normalized to the QC sample using support vector regression (SVR) from the R package MetNormalizer (Shen et al., 2016) in the primary analyses of both QC and tested samples to ensure high‐quality data within an analytical run. This step has been broadly accepted as a strategy assuring the data quality of metabolite profiles. The relative standard deviation (RSD) value of metabolites in the QC samples was set at the threshold of 30% as the standard in the assessment of the repeatability of metabolome data sets.

### Targeted metabolites profiling

Targeted determination and quantification of selected metabolites were performed as described previously (Liu et al., 2017). We measured 108 metabolites including 21 metabolites of bile acid metabolism, 29 metabolites of amino acid metabolism, 20 metabolites of organic acid metabolism, 18 metabolites of nucleotide metabolism, 9 metabolites of carboxylic acid metabolism, 3 metabolites of lipids metabolism, 7 vitamins and 1 choline. In brief, 100 μL of serum samples was blended with internal standard mixed solutions that contained four deuterium‐labelled BAs (CA‐d4, CDCA‐d4, LCA‐d4, and GCA‐d4) purchased from Steraloids (Newport, USA) and five other deuterium‐labelled BAs (DCA‐d4, GCDCA‐d4, GDCA‐d4, UDCA‐d4 and GLCA‐d4) purchased from Cambridge Isotope Laboratories (Tewksbury). In addition, the standard mixed solutions diluted at various concentrations were spiked into isotope‐labelled standard solutions to prepare calibration curves for the quantification of serum metabolites. Briefly, 100 μL of serum sample was deproteinized with 400 μL methanol (Merck, Germany) at −20°C for 20 min. Then, 400 μL of supernatant was freeze‐dried. One hundred microliters of acetonitrile‐water (1:1) was added to the freeze‐dried residues and vortexed. The mixture was centrifuged and the supernatant was submitted for measurement.

The measurements of targeted metabolites were performed by a UPLC coupled to a SCIEX 5500 QTRAP mass spectrometry (SCIEX) with the ESI source. An ACQUITY UPLC C18 column (1.7 μm × 2.1 μm × 100 mm) (Waters, USA) was used to separate bile acid compounds with a flow rate of 0.3 mL/min at a column temperature of 45°C. Eluent A was formed by water with 0.1% formic acid, and methanol was used as eluent B. The linear gradient of column elution was set as follows: 60%–65% B at 0–6 min, 65%–80% B at 6–13 min, 80%–90% B at 13–13.5 min, and 95% B at 13.5–15 min. All samples were kept at 8°C during detection. The source parameters were set as follows: capillary voltage, −4.5 kV; source temperature, 550°C; ion source gas 1, 55; ion source gas 2, 55; and curtain gas, 40.

The instruments, chromatographic column and flow rate for quantifying the other 87 metabolites were as same as the bile acid derivates described above. The column temperature was set at 45°C. Eluent A was formed by water with 25 mM ammonium acetate and 25 mM ammonia, and acetonitrile was used as eluent B. The linear gradient of column elution was set as follows: 95% B at 0–1 min, 95%–65% B at 1–14 min, 65%–40% B at 14–16 min, 40% B at 16–18 min, 40–95% B at 18–18.1 min and maintained for 4.9 min. All 30 samples were kept at 8°C during the detection. The source parameters were set as follows: sheath gas temperature, 350°C; dry gas temperature, 350°C; sheath gas flow, 11 L/min; dry gas flow, 10 L/min; capillary voltage, 4000 V and 3500 V in positive and negative modes, respectively; nozzle voltage, 500 V; and nebulizer pressure, 30 psi. The dwell time of each Multiple Reaction Monitoring (MRM) ion pair was 3 ms, and the total cycle time was 1.263 s.

### Statistical analysis

#### Pairwise microbiota‐metabolite association analysis and the contribution of gut microbiota to the variance of serum metabolites

Samples with both 16S rRNA gene sequencing and serum metabolome data were used to analyse the associations between gut microbiota and serum metabolites for each of three gut locations (Figure S1A). Before the association analyses, we first filtered those genera and ASVs with relative abundance <0.05% and presented <5% of tested samples. Considering that the distribution of the abundances of ASVs or taxa was not a fitted normal distribution, the association analyses between gut microbiota and serum metabolites, and the contribution of gut microbiota to the variation of serum metabolites were calculated by a two‐part model that was based on the linear model (Fu et al., 2015). The fixed effects of sex and slaughter batch could be adjusted. The model accounted for both binary and quantitative features of microbial data. In brief, the two‐part model includes a binary model and a quantitative model. The binary model accounted for the effect of the presence/absence (1 for detected or 0 for undetected) of a microbial taxonomy on serum metabolites, while the quantitative model was used to evaluate the association between the abundance of the microbes and serum metabolite values. To further identify whether the correlation came from the presence/absence or the abundance of the gut microbiota or both, the combination of the binary and quantitative analysis was characterized by a meta‐analysis in which the *p*‐value was derived from an unweighted Z score method. Correlation analyses were performed at both genus and ASV levels. To evaluate the percentage of the variation of microbial‐associated metabolites explained by the gut microbiota, we conducted a 100 times cross‐validation with the two‐part model as we previously reported (He et al., 2016).

#### Comparison of the α‐ and β‐ diversity of microbial communities

The α‐diversity of the microbial community including observed features and Shannon index was compared pairwise among three gut locations by the Wilcoxon rank‐sum test. Principal component analysis (PCA) was performed to show the discrepancy of the phylogenetic compositions of gut microbiota among different gut locations by the FactoMineR package in R (4.0.2).

#### Pairwise correlations between targeted serum metabolites and gut microbiota

After quality control based on the partial least square discriminant analysis (PLS‐DA), the datasets from two experimental pigs failed to pass quality control and were removed from further analyses. The correlation analysis between targeted serum metabolites and microbial taxa was first performed by the two‐part model as described above for untargeted metabolites. Moreover, considering the small sample size (28 samples), the correlations between targeted serum metabolites and microbiota taxa were also tested by Spearman rank correlation analysis. The fixed effects including sex and slaughter batch were identified and adjusted by *pcor* function in R. Spearman correlation analyses were performed at the genus, species and ASV levels.

We retained the correlation results fitting the following two criteria: (i) the metabolites could be annotated based on the HMDB database with MS/MS fragmentation and satisfied the annotation requirements of Level 1 and Level 2 from the MSI; (ii) significant associations were found between bacterial genera and metabolites or between ASVs and metabolites. *p*‐values obtained from the association analyses by the two‐part model were adjusted for the multiple tests (FDR < 0.05), while *p*‐values of Spearman correlations were adjusted by the Benjamini‐Hochberg (BH) method. Network‐based enrichment analysis of serum metabolites was conducted by the R package FELLA. All statistical analyses were performed in R (v4.0.2).

## AUTHOR CONTRIBUTIONS

**Qin Liu:** Conceptualization (equal); formal analysis (equal); investigation (equal); writing – original draft (equal); writing – review and editing (equal). **Maozhang He:** Conceptualization (equal); formal analysis (equal); investigation (equal); visualization (equal); writing – original draft (equal); writing – review and editing (equal). **Zhijun Zeng:** Data curation (equal); visualization (equal); writing – review and editing (equal). **Xiaochang Huang:** Data curation (equal); writing – review and editing (equal). **Shaoming Fang:** Investigation (equal); methodology (equal). **YuanZhang Zhao:** Data curation (equal); investigation (equal); visualization (equal). **Shanlin Ke:** Data curation (equal); methodology (equal). **Jinyuan Wu:** Data curation (equal); investigation (equal); methodology (equal). **Yunyan Zhou:** Data curation (equal); investigation (equal); methodology (equal). **Xinwei Xiong:** Data curation (equal); investigation (equal); methodology (equal); visualization (equal). **Zhuojun Li:** Data curation (equal); formal analysis (equal); methodology (equal); visualization (equal). **Hao Fu:** Data curation (equal); writing – review and editing (equal). **Lusheng Huang:** Conceptualization (equal); formal analysis (equal); investigation (equal); visualization (equal); writing – original draft (equal); writing – review and editing (equal). **Congying Chen:** Conceptualization (equal); formal analysis (equal); project administration (equal); supervision (equal); visualization (equal); writing – original draft (equal); writing – review and editing (equal).

## CONFLICT OF INTEREST STATEMENT

The authors declare no competing interests.

## ETHICS STATEMENT

All animal works were conducted according to the guidelines for the care and use of experimental animals established by the State Council of the People's Republic of China (Decree No. 2, 1988). This study was also approved by the Animal Care and Use Committee (ACUC) of Jiangxi Agricultural University (No. JXAU2011‐006).

## Supporting information


Figure S1.
Figure S2.
Figure S3.
Figure S4.

Table S1.
Table S2.
Table S3.
Table S4.
Table S5.
Table S6.
Table S7.
Table S8.
Table S9.
Table S10.
Table S11.
Table S12.
Table S13.
Table S14.

## Data Availability

All 16S rRNA gene sequencing data were submitted to the GSA database with accession number: CRA006230.
