# Supplementary material for: Extensive identification of serum metabolites related to microbes in different gut locations and evaluating their associations with porcine fatness
Source: Microb Biotechnol. 2023 Mar 14;16(6):1293–311. doi: 10.1111/1751-7915.14245 (PMC10221527; doi:10.1111/1751-7915.14245)
Supplement: Supplementary file 1 — Figure S1. Figure S2. Figure S3. Figure S4. [file MBT2-16-1293-s001.docx]

**Supplementary Information**

**Extensive** **identification of serum metabolites related to microbes in different gut locations and evaluating their associations with porcine fatness**

**Qin Liu^1#^,** **Maozhang He^1,2 #^, Zhijun Zeng^3^,** **Xiaochang Huang****^1^, Shaoming Fang^1^, Yuanzhang Zhao^1^, Shanlin Ke^1^, Jinyuan Wu^1^, Yunyan Zhou^1^, Xinwei Xiong^1^, Zhuojun Li^1^, Hao Fu^1^, Lusheng Huang^1*^, Congying Chen^1*^**

^1^National Key Laboratory for Swine Genetic Improvement and Production Technology, Jiangxi Agricultural University, Nanchang 330045, China;

^2^Department of Microbiology, School of Basic Medical Sciences, Anhui Medical University, Hefei 230032, China;

^3^Research Center for Differention and Development of TCM Basic Theory, Jiangxi Province Key Laboratory of TCM Etiopathogenisis, Jiangxi University of Traditional Chinese Medicine, Nanchang 330004, China.

^#^Both Qin Liu and Maozhang He contributed equally to this work.

^*^**Corresponding authors**: Congying Chen, Lusheng Huang.

National Key Laboratory for Swine Genetic Improvement and Production Technology, Jiangxi Agricultural University, Nanchang, 330045, Jiangxi, China;

Email: chcy75@hotmail.com;

Lushenghuang@hotmail.com.

Tel: 0086-791-83813080; Fax: 0086-791-83900189.

Supplementary Figure 1-4.


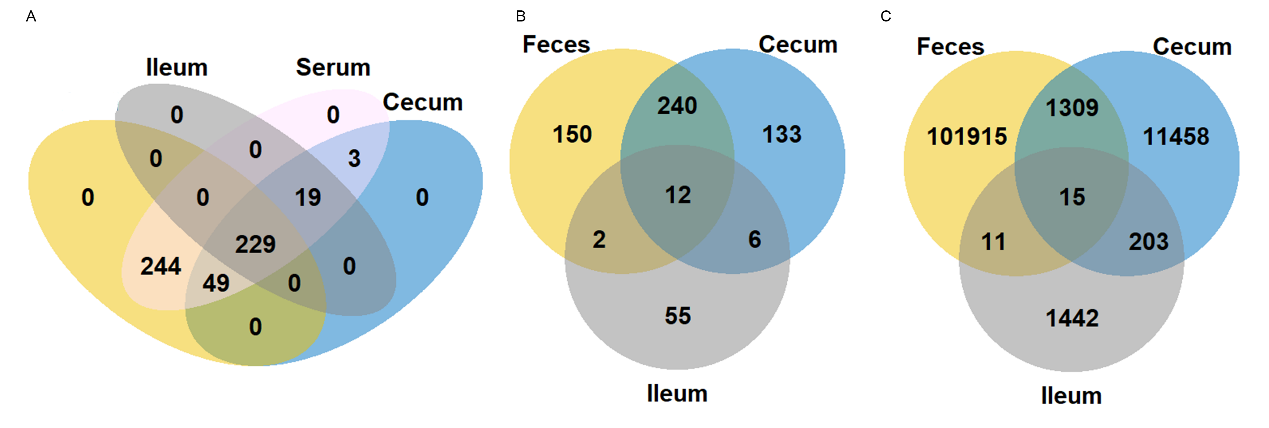


**Supplementary Figure 1. Venn diagrams showing the numbers of samples, ASVs, and significant correlations shared across gut locations or specific to each gut location.** (A) The numbers of microbial samples used for 16S RNA gene sequencing from three gut locations, and serum samples used for untargeted metabolome measurement. (B) The numbers of ASVs shared across gut locations or specific to each gut location. (C) The numbers of significant correlations shared across gut locations or specific to each gut location.


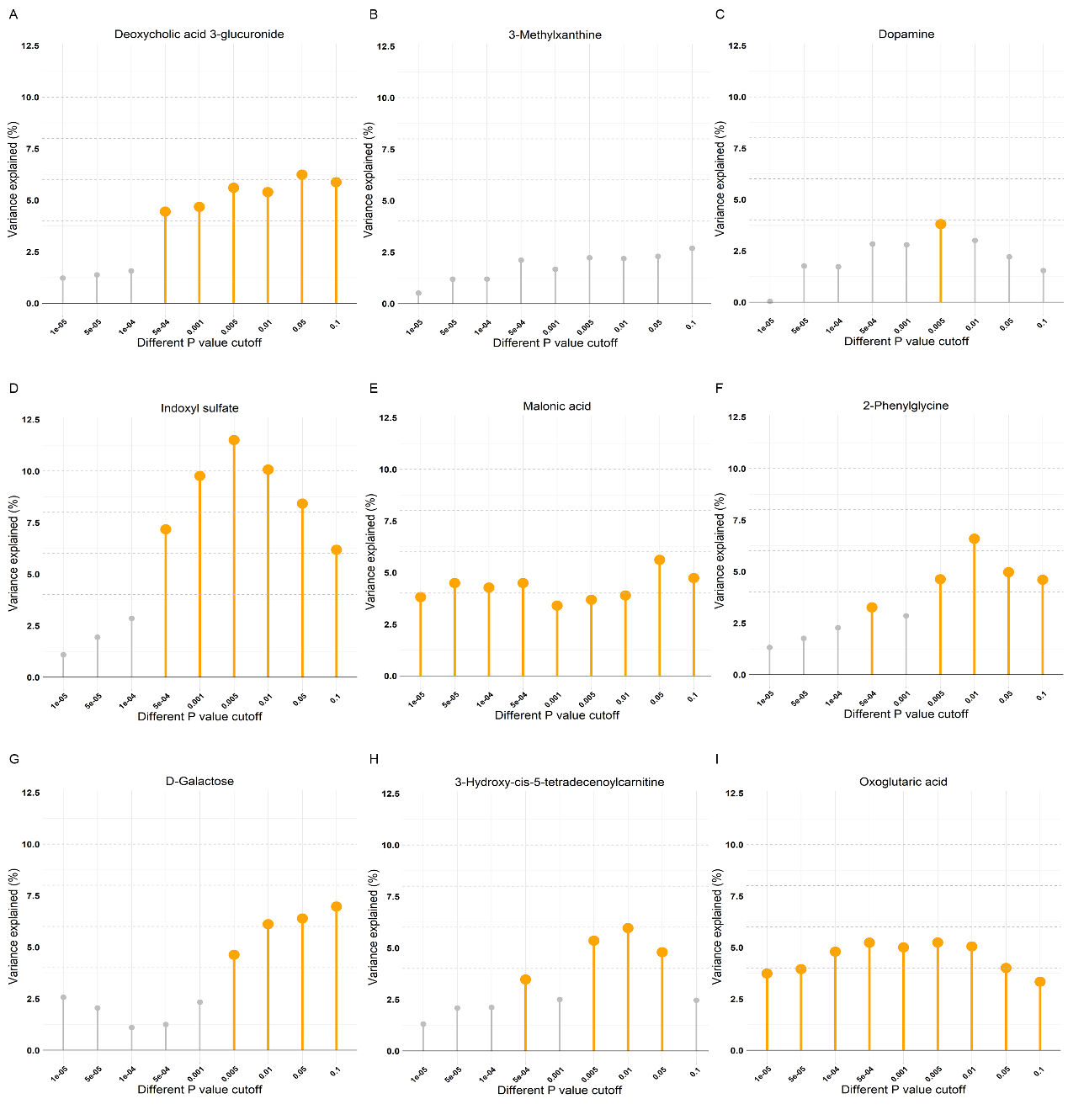


**Supplementary Figure 2.** **The contribution of gut microbiota to the variations of serum metabolites.** The metabolites whose variation in tested samples explained by the gut microbiota were ranked in the top three in the ileum (A-C), cecum (D-F) and stool (G-I). (A-C) The contribution of gut microbiota to the variations of deoxycholic acid 3-glucuronide, 3-Methylxanthine, and dopamine in ileum samples. (D-F) The contribution of gut microbiota to the variations of indoxyl sulfate, malonic acid, and 2-phenylglycine in cecum samples. (G-I) The contribution of gut microbiota to the variations of D-galactose, 3-hydroxy-cis-5-tetradecenoylcarnitine, and oxoglutaric acid in fecal samples. The orange edges indicate the variation proportion of gut microbiota exceed 30%.


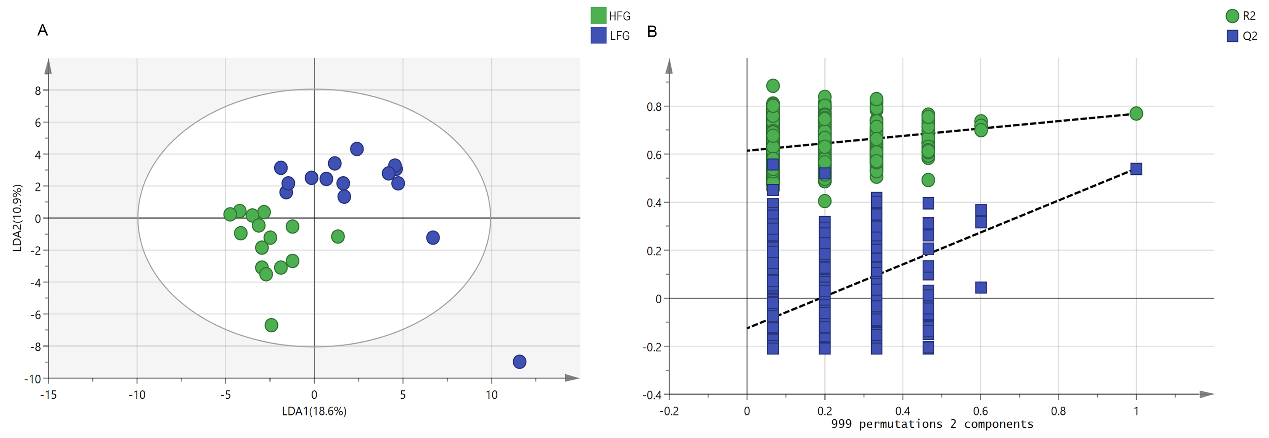


**Supplementary Figure 3. Serum metabolome profiles between high-fatness group (HFG) and low-fatness group (LFG).** (A) PLS-DA score plot shows significant difference of serum metabolite profiles between HFG and LFG groups. (B) Validated plot of response permutation testing. The Y-axis represents R^2^Y (triangles) and Q^2^Y (squares) for PLS-DA model. The X-axis indicates the correlation coefficient between original data and permuted response data.


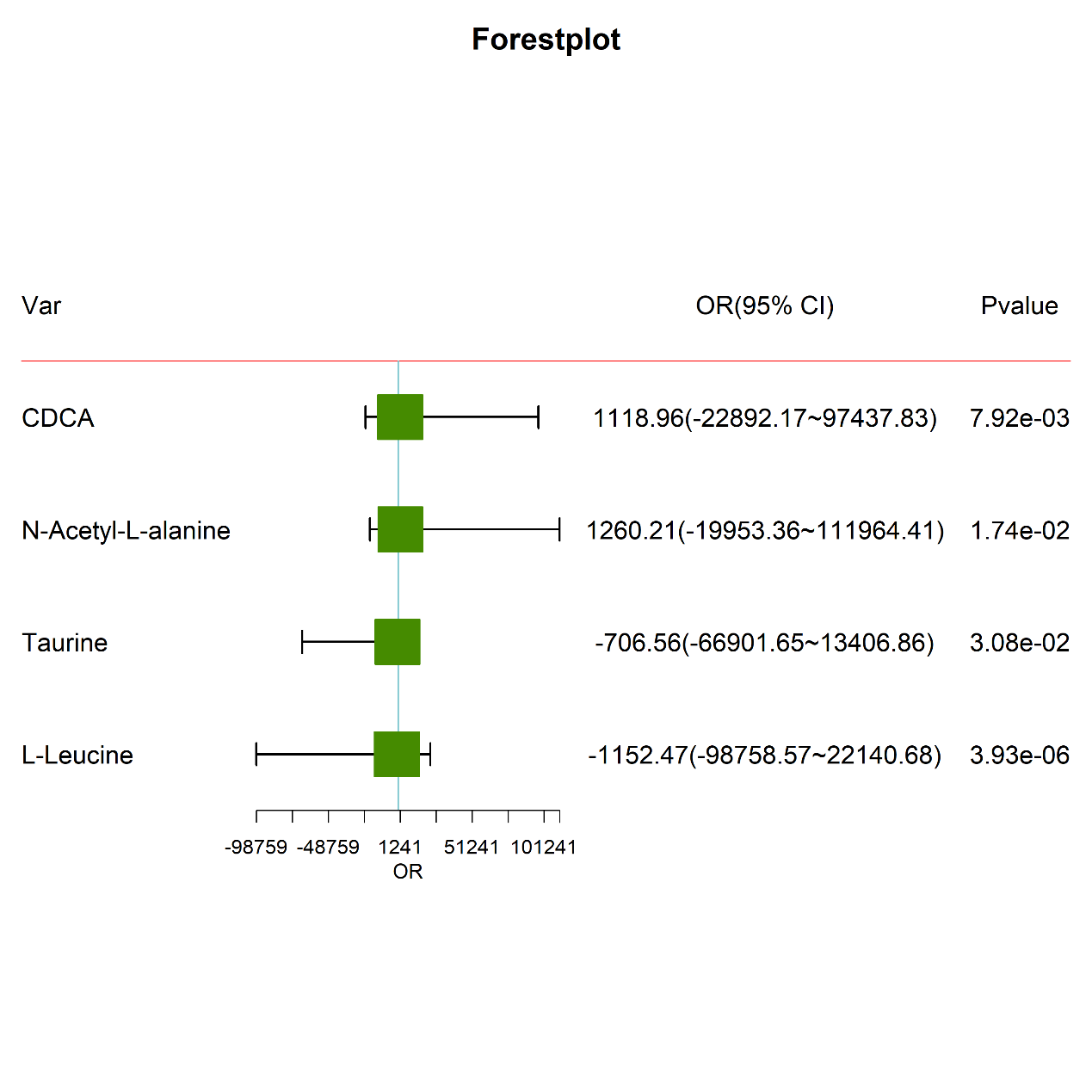


**Supplementary Figure 4. Forest plot of four serum metabolites as potential biomarkers for predicting pig fat deposition.** Odds ratios (ORs) and corresponding 95% confidence intervals for four serum metabolites in the high-fatness pig group (HFG) and low-fatness pig (LFG) group. Var means variates, and CI means confidence interval.

Supplementary Tables 1-14.

Table S1: Serum metabolites identified by the untargeted metabolome measurement.

Table S2: Significant correlations between gut microbial genera in the ileum and serum metabolites.

Table S3: Significant correlations between gut microbial ASVs in the ileum and serum metabolites.

Table S4: Significant correlations between gut microbial genera in the cecum and serum metabolites.

Table S5: Significant correlations between gut microbial ASVs in the cecum and serum metabolites.

Table S6: Significant correlations between gut microbial genera in feces samples and serum metabolites.

Table S7: Significant correlations between gut microbial ASVs in feces samples and serum metabolites.

Table S8: Significant correlations between gut microbial ASVs in three gut locations and serum metabolites identified by spearman rank correlation analysis.

Table S9: Significant correlations shared across three gut locations.

Table S10: Significant correlations identified in both ileum and cecum samples.

Table S11: Significant correlations identified in both cecum and fecal samples.

Table S12: The variations of serum metabolites in the tested samples explained by the ileal microbiota at different cutoff of P-values.

Table S13: The variations of serum metabolites in the tested samples explained by the cecal microbiota at different cutoff of P-values.

Table S14: The variations of serum metabolites in the tested samples explained by the fecal microbiota at different cutoff of P-values.
